# The IntAct database: efficient access to fine-grained molecular interaction data

**DOI:** 10.1093/nar/gkab1006

**Published:** 2021-11-11

**Authors:** Noemi del Toro, Anjali Shrivastava, Eliot Ragueneau, Birgit Meldal, Colin Combe, Elisabet Barrera, Livia Perfetto, Karyn How, Prashansa Ratan, Gautam Shirodkar, Odilia Lu, Bálint Mészáros, Xavier Watkins, Sangya Pundir, Luana Licata, Marta Iannuccelli, Matteo Pellegrini, Maria Jesus Martin, Simona Panni, Margaret Duesbury, Sylvain D Vallet, Juri Rappsilber, Sylvie Ricard-Blum, Gianni Cesareni, Lukasz Salwinski, Sandra Orchard, Pablo Porras, Kalpana Panneerselvam, Henning Hermjakob

**Affiliations:** European Bioinformatics Institute (EMBL-EBI), European Molecular Biology Laboratory, Hinxton, Cambridgeshire CB10 1SD, UK; European Bioinformatics Institute (EMBL-EBI), European Molecular Biology Laboratory, Hinxton, Cambridgeshire CB10 1SD, UK; European Bioinformatics Institute (EMBL-EBI), European Molecular Biology Laboratory, Hinxton, Cambridgeshire CB10 1SD, UK; European Bioinformatics Institute (EMBL-EBI), European Molecular Biology Laboratory, Hinxton, Cambridgeshire CB10 1SD, UK; Wellcome Centre for Cell Biology, University of Edinburgh, Edinburgh EH9 3BF, UK; European Bioinformatics Institute (EMBL-EBI), European Molecular Biology Laboratory, Hinxton, Cambridgeshire CB10 1SD, UK; European Bioinformatics Institute (EMBL-EBI), European Molecular Biology Laboratory, Hinxton, Cambridgeshire CB10 1SD, UK; Fondazione Human Technopole, Milan 20157, Italy; UCLA-DOE Institute for Genomics and Proteomics, University of California, Los Angeles, CA 90095, USA; UCLA-DOE Institute for Genomics and Proteomics, University of California, Los Angeles, CA 90095, USA; UCLA-DOE Institute for Genomics and Proteomics, University of California, Los Angeles, CA 90095, USA; UCLA-DOE Institute for Genomics and Proteomics, University of California, Los Angeles, CA 90095, USA; Gibson Group, European Molecular Biology Laboratory, Heidelberg 69117, Germany; European Bioinformatics Institute (EMBL-EBI), European Molecular Biology Laboratory, Hinxton, Cambridgeshire CB10 1SD, UK; European Bioinformatics Institute (EMBL-EBI), European Molecular Biology Laboratory, Hinxton, Cambridgeshire CB10 1SD, UK; Bioinformatics and Computational Biology Unit, Dept. of Molecular Biology, University of Rome Tor Vergata, Rome, Italy; Bioinformatics and Computational Biology Unit, Dept. of Molecular Biology, University of Rome Tor Vergata, Rome, Italy; Department of Molecular, Cell and Developmental Biology, University of California, Los Angeles, CA 90095, USA; European Bioinformatics Institute (EMBL-EBI), European Molecular Biology Laboratory, Hinxton, Cambridgeshire CB10 1SD, UK; Dipartimento di Biologia, Ecologia e Scienze della Terra, Università della Calabria, Rende, Italy; European Bioinformatics Institute (EMBL-EBI), European Molecular Biology Laboratory, Hinxton, Cambridgeshire CB10 1SD, UK; UCLA-DOE Institute for Genomics and Proteomics, University of California, Los Angeles, CA 90095, USA; ICBMS UMR CNRS 5246, University Lyon 1, Lyon, Villeurbanne 69622, France; Wellcome Centre for Cell Biology, University of Edinburgh, Edinburgh EH9 3BF, UK; Bioanalytics, Institute of Biotechnology, Technische Universität Berlin, Berlin 13355, Germany; ICBMS UMR CNRS 5246, University Lyon 1, Lyon, Villeurbanne 69622, France; Bioinformatics and Computational Biology Unit, Dept. of Molecular Biology, University of Rome Tor Vergata, Rome, Italy; UCLA-DOE Institute for Genomics and Proteomics, University of California, Los Angeles, CA 90095, USA; European Bioinformatics Institute (EMBL-EBI), European Molecular Biology Laboratory, Hinxton, Cambridgeshire CB10 1SD, UK; European Bioinformatics Institute (EMBL-EBI), European Molecular Biology Laboratory, Hinxton, Cambridgeshire CB10 1SD, UK; European Bioinformatics Institute (EMBL-EBI), European Molecular Biology Laboratory, Hinxton, Cambridgeshire CB10 1SD, UK; European Bioinformatics Institute (EMBL-EBI), European Molecular Biology Laboratory, Hinxton, Cambridgeshire CB10 1SD, UK

## Abstract

The IntAct molecular interaction database (https://www.ebi.ac.uk/intact) is a curated resource of molecular interactions, derived from the scientific literature and from direct data depositions. As of August 2021, IntAct provides more than one million binary interactions, curated by twelve global partners of the International Molecular Exchange consortium, for which the IntAct database provides a shared curation and dissemination platform. The IMEx curation policy has always emphasised a fine-grained data and curation model, aiming to capture the relevant experimental detail essential for the interpretation of the provided molecular interaction data. Here, we present recent curation focus and progress, as well as a completely redeveloped website which presents IntAct data in a much more user-friendly and detailed way.

## INTRODUCTION

Biomolecular interactions are the fabric underlying almost all processes in living organisms, and they are determined by a broad array of experimental approaches, from focussed studies of pairwise interactions to large-scale determination of 10 000s of interactions in standardised high throughput experiments. However, observed molecular interactions are highly dependent on the biological and experimental conditions under which they are determined. Cellular systems, experimental protein tags sequence modifications, and experimental approaches all heavily influence the observed interaction. Since its inception in 2005, members of the International Molecular Exchange Consortium (IMEx) (1) have collaboratively curated molecular interaction data from the scientific literature and from direct data depositions, emphasizing a deep curation model aiming to capture interaction reports in sufficient detail to support subsequent comprehensive data presentation, aggregation, and analysis. In 2017, the IMEx Consortium became an ELIXIR core data resource (2), recognising it as part of the fundamental infrastructure for life sciences. For an in-depth review of the current IMEx data model, curation strategies and collaborations, see (3). The IntAct database of molecular interactions is used by all currently active IMEx partners (IntAct, DIP (4), UniProt (5), MINT (6), MatrixDB (7), UCL ICS, IID (8)) as a common curation platform, and also acts as a common data dissemination platform, in parallel to the partners’ own websites. While the detailed IMEx interaction data has always been available through download in the feature-rich PSI-MI XML format (9,10), many annotation details were not conveniently accessible through the IntAct website, and often users are not aware of the depth of available annotations. We are increasingly addressing this issue through the release of targeted datasets, in particular for sequence variations impacting interactions, and through a completely redeveloped website, which provides comprehensive filter and display tools to make optimal use of the rich annotation available in the IntAct database.

### Data Content

Since the last IntAct NAR publication (11), data content has grown from 408 000 (Jamuary 2014) to 1 114 500 (June 2021)) interaction evidences, and the number of referenced publications has risen from 12 500 to 22 500. This rapid increase is based on the integration of previously curated data from IMEx partners, as well as the ongoing curation work. The faster rise in interaction numbers compared to publication numbers reflects the increasing trend towards large-scale interaction studies. In the same period, interactions from 21 publications have been retracted, usually due to retraction of the supporting publication. Several new datasets have been released, including two key collections: the ‘Mutations dataset’ (6) and the ‘Coronavirus interactome’ (7).

### Mutations dataset

This dataset contains annotations describing the effect of small sequence changes on protein interactions. Captured changes comprise both natural variants and experimentally introduced sequence changes. This dataset is continuously maintained and updated, and since the original publication in February 2019 (12) it has grown from 28 000 to 72 000 mutation annotations. In order to fully reflect the importance of this data and to improve accessibility to it for users, in addition to web interface changes (see below), we have also introduced a dedicated tab-delimited download file format (https://www.ebi.ac.uk/intact/download/datasets#mutations).

### Coronavirus interactome

After the outbreak of the COVID19 pandemic in Europe in early March 2020, we initiated an IMEx-wide initiative to record molecular interaction data related to SARS-CoV-2 and other members of the Coronaviridae family of viruses, along with human protein interactions of potential relevance for the disease's aethiopathology. Since its publication in November 2020 (13), the Coronavirus interactome dataset has grown from 4400 interaction evidences derived from 151 publications to 9100 interaction evidences from 332 publications in June 2021, and is accessible at https://www.ebi.ac.uk/intact/resources/datasets#coronavirus. Work is still actively ongoing to capture novel interactions, and details of known interactions such as the effects of variants, to further enhance this dataset.

### Curation Policies

Given the fast pace at which COVID19-related data has been generated, the IMEx Consortium decided to allow the curation of preprints when the scientific interest contained in these publications justifies it. We will periodically review and update these datasets to ensure only data from peer-reviewed publications is maintained in the database long-term. More information about IMEx's curation policy regarding preprints is provided at www.imexconsortium.org/curation/.

Curation practices and controlled vocabularies/ontologies are continuously updated, driven by the development of new methods like BioID (14) (term in PSI-MI Ontology https://www.ebi.ac.uk/ols/ontologies/mi/terms?iri = http%3A%2F%2Fpurl.obolibrary.org%2Fobo%2FMI_1314). The IntAct sibling resource, the Complex Portal (15), now provides a reference resource for biomolecular complexes, and we are annotating complexes with Complex Portal identifiers as interacting objects where possible, in addition to interactions of proteins with small molecules, nucleic acids and polysaccharides such as glycosaminoglycans.

### Web Site

**Figure 1.**
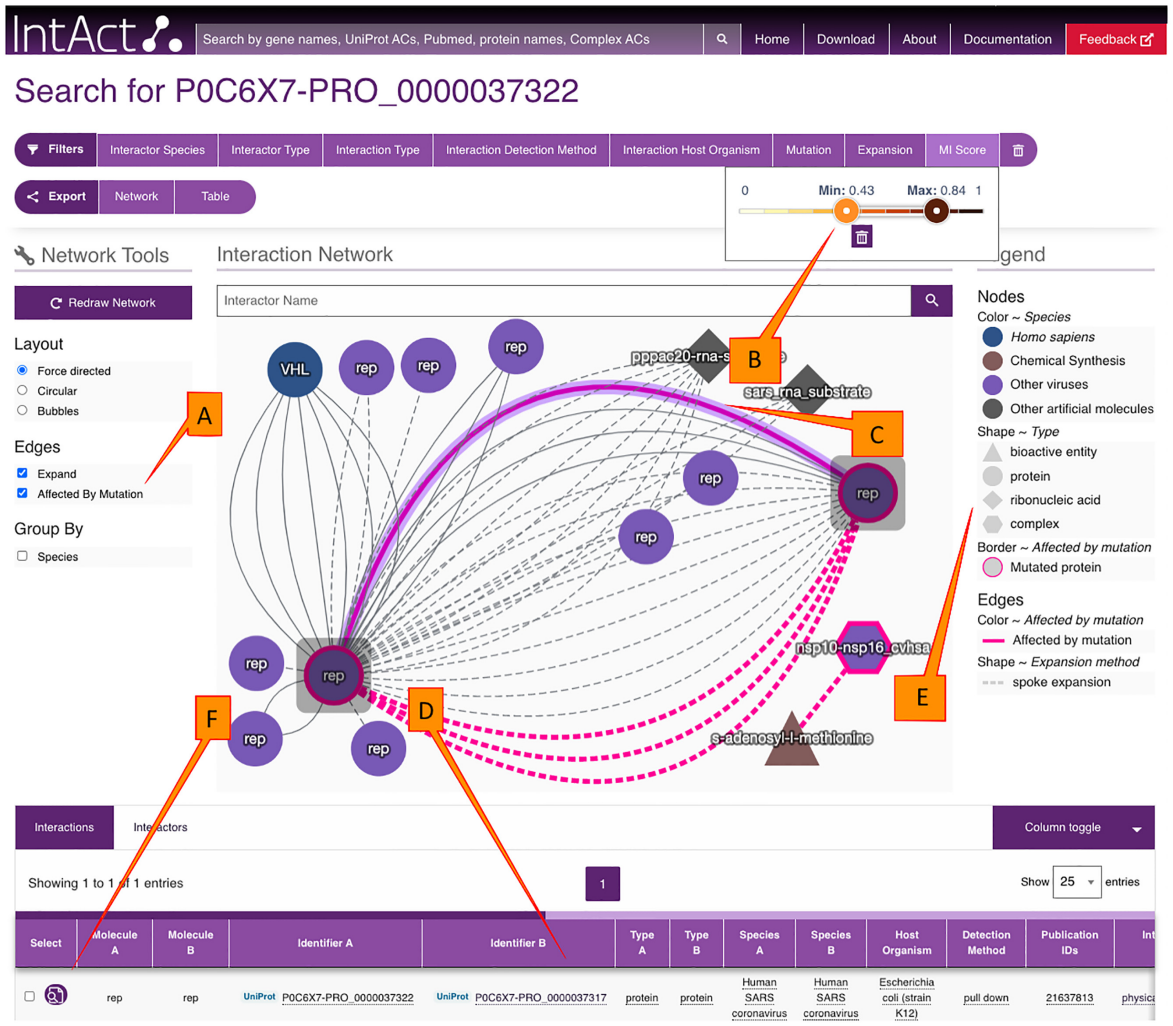
IntAct search results for UniProtKB:P0C6 × 7-PRO_0000037322. The option ‘Affected by Mutation’ has been selected in facet (**A**), highlighting corresponding interactions in bold pink. The minimum MI score (label **B**) has been set to 0.43. One interaction between SARS-CoV proteins, nsp10-nsp16 is highlighted (through mouse click) in purple (edge **C**). The interaction table (**D**) automatically shows only the highlighted interaction. The legend on the right (**E**) documents the representation of species, type of biomolecule, mutations and edge types. Clicking on the magnifying glass (icon **F**) provides detailed information on an interaction, as shown in Figure 2. Interactor positions can be manually rearranged through drag-and-drop, as done here. Figure is a modified screen capture from.https://www.ebi.ac.uk/intact/search?query=P0C6X7-PRO_0000037322&minMIScore=0.43&mutationStyle=true.

**Figure 2.**
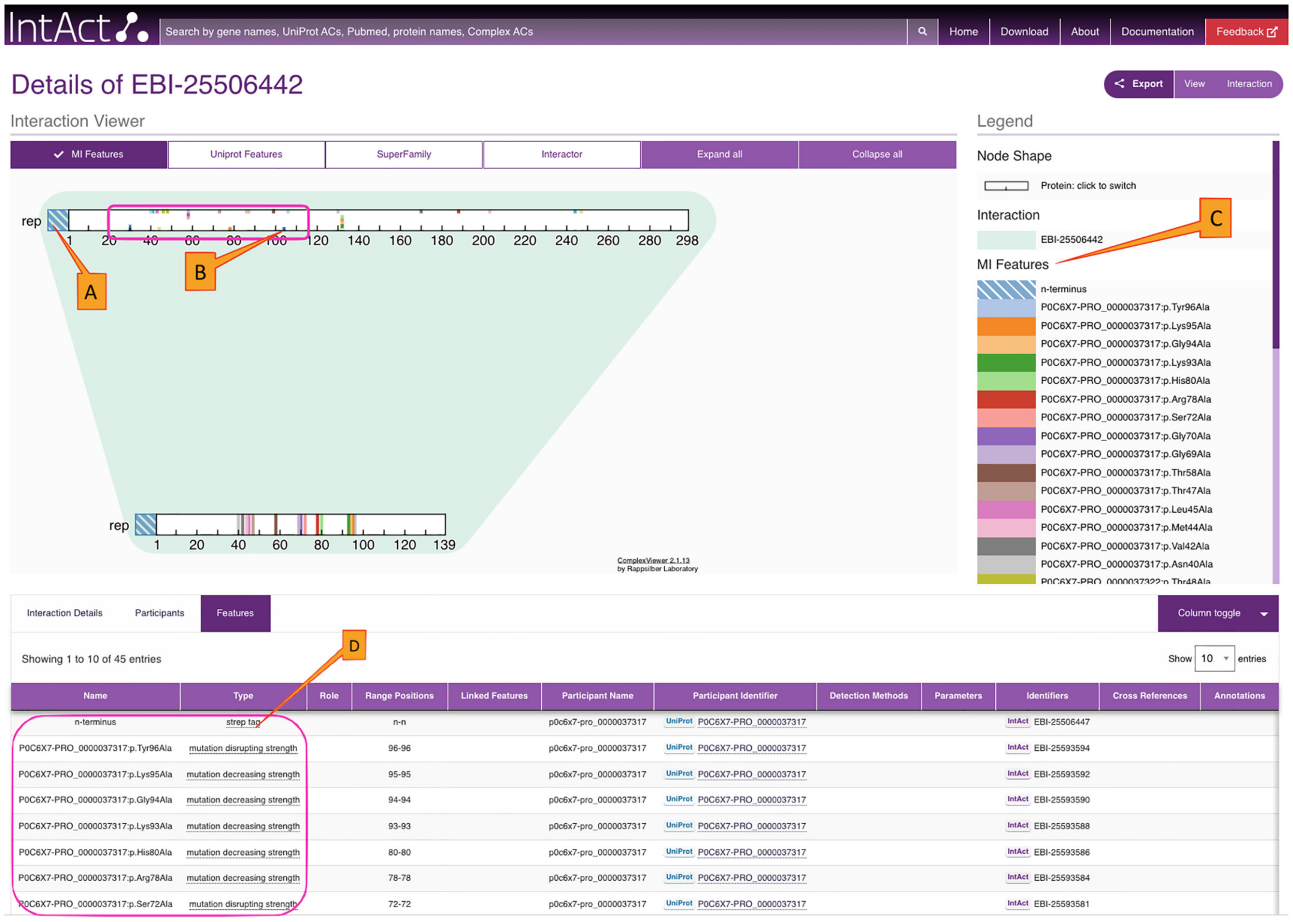
This figure shows the interaction viewer for the highlighted edge from Figure 1. Features of the participants including the N-terminal tags (**A**) and all the mutations annotated for this interaction are displayed in the viewer (**B**) and also in the legend (**C**). All the features are mapped at the amino acid level of the proteins. Further details on the features are available from the features tab (**D**) below the Interaction viewer. Figure is a modified screen capture from https://www.ebi.ac.uk/intact/details/interaction/EBI-25506442.

**Figure 3.**
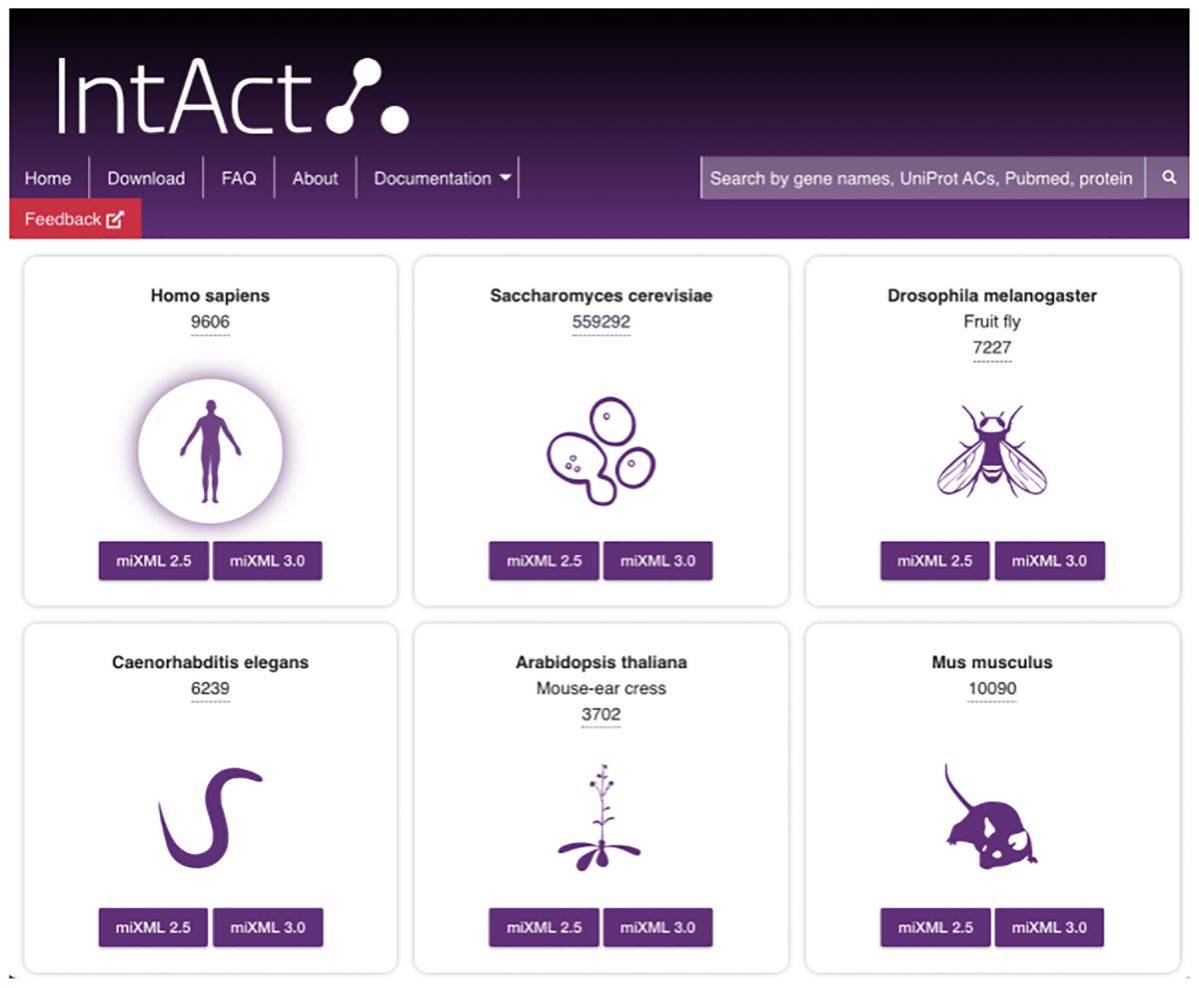
Species-specific interactomes are easily accessible from the ‘Interactomes’ tile of the home page.

We have redeveloped the IntAct web site (https://www.ebi.ac.uk/intact) to provide efficient, user-friendly access to IntAct data content, with a focus on filter and display functionality to make the detailed interaction data accessible and useful through the user interface. The quick search provides autocompletion to facilitate selection of molecules of interest based on gene names, protein names, and accession numbers. The batch search supports multiple simultaneous query terms and subsequent result refinement. Results are shown both graphically and in tabular format, can be modified through comprehensive filter and visualisation options, and exported in both tabular and graphical formats. Figure 1 provides a view of the new IntAct web interface and its functionality, Figure 2 demonstrates the high level of detail provided for a single interaction. In addition to queries, species-specific interactomes (Figure 3) and datasets like ‘Alzheimers’ (16) are available from tiles on the home page.

### Implementation

The new IntAct public instance is deployed on the EMBL-EBI cloud using Kubernetes to manage the different containerized applications (the images have been built with Docker). The IntAct public interface is based on a Neo4j graph database and Apache Solr to enable the search and navigation features. An externally accessible API (https://www.ebi.ac.uk/intact/documentation/technical_corner#apis), developed in Java™ with the Spring framework to ease the implementation of the microservices architecture, serves data to both the web application and the Cytoscape app (17). The web frontend is a single page application implemented with the Angular framework together with the EMBL-EBI Visual framework for general styling (https://www.ebi.ac.uk/style-lab/websites/). The network display (Figure 1) is based on Cytoscape.js (18), the interaction detail view is the ComplexViewer (19). The IntAct web-based user interface has been specified using two rounds of user testing based on mockups. To provide a consistent user experience, we are co-ordinating the visualisation of interacting molecules in terms of shape (for molecule types) and default colour (for species) between the IntAct web interface and the IntAct Cytoscape app.

### Perspectives

#### Tissue specificity

Recent research emphasizes fundamental differences among cell type specific interactomes (20). Detailed annotation of cell types/tissue has been standard practice in IMEx curation for a long time, but the information is currently partially in free text form and will benefit from standardisation and integration with ontologies like Experimental Factor Ontology (21), Brenda (22), Uberon (23), Cell Line Ontology (24), and Cellosaurus (25). We are currently working on the restructuring of cell type/tissue annotation and increasing exposure of these data through download files and user interfaces.

#### Rare diseases dataset

As part of our commitment to the clinical community, we are currently populating a rare disease dataset, with a focus on interactions affected by rare disease mutations. Approximately 5500 rare disease interactions have been annotated to date, assigning details such as kinetic parameters, variable experimental conditions or construct details, including binding surfaces and mutations that affect the interactions. The data features information about the amino acid changes, their effect over the interaction and full reference to the experimental interaction evidence from which it was extracted. Currently, around 98% of the annotations are mapped to human proteins, providing high-quality experimental evidence of sequence change effects which directly relate to existing variation data.

#### Credit attribution

The data presented here has been carefully curated over almost two decades by professional curators from twelve IMEx partners. To value scientific database curation as a key scientific activity in its own right, we are working on credit attribution for past and future IMEx curators through APICURON (26) and ORCID (https://orcid.org/).

### Box text: key concepts

IMEx: The International Molecular Exchange Consortium (IMEx), founded in 2005, is an international collaboration of twelve interaction data resources which coordinate their curation strategies. IntAct is an IMEx founding member, and provides the web-based curation platform used by all current IMEx partners.

Interaction evidence: Interactions may have two or more participating molecules, and the number of observable interactors may depend on both biological and experimental constraints. As an example, the yeast-two-hybrid array technology (27) typically identifies only pairs of interactors (binary interactions), while techniques like tandem affinity purification (TAP) (28) and BioID (29) may identify two or more interacting molecules (*n*-ary interactions). Observed *n*-ary interactions are stored as such in the IntAct database, but for some download files and for visualisation, counting and comparison purposes, they are expanded into multiple binary interactions. In addition, one publication may use more than one experimental method to determine an interaction. One interaction evidence is one pair of interacting molecules, observed by one experimental approach, reported by one publication. In this manuscript, we use ‘interaction’ as a synonym for the technically more correct term of ‘interaction evidence’.

MI Score: The MI Score (30) is a quantitative estimate of the confidence in a given interaction. It is a normalized and weighted count of independent interaction evidence and associated experimental methods.

## DATA AVAILABILITY

IntAct is open source, open data. The source code is available from https://github.com/intact-portal, all data is freely available through the web interface, API, and from https://www.ebi.ac.uk/intact/download under the CC BY 4.0 licence.
